# Association between atherogenic index of plasma, body mass index, and sarcopenia: a cross-sectional and longitudinal analysis study based on older adults in China

**DOI:** 10.1007/s40520-025-03029-0

**Published:** 2025-04-07

**Authors:** Bowen Lu, Jiacheng Li, Xuezhen Liang, Mingtao Wen, Di Luo, Haifeng Jia, Jiahao Zhang, Gang Li

**Affiliations:** 1 The First Clinical Medical School, Shandong University of Traditional Chinese Medicine, Jinan, Shandong China; 2 Orthopaedic, Affiliated Hospital of Shandong University of Traditional Chinese Medicine, Jinan, Shandong China

**Keywords:** Sarcopenia, Atherogenic index of plasma (AIP), Body mass index (BMI), CHARLS database, Older adults

## Abstract

**Objective:**

To investigate the correlation between the atherogenic index of plasma (AIP), body mass index (BMI), and sarcopenia in the older adults in China, and to analyze the predictive ability of AIP and BMI for sarcopenia.

**Methods:**

This study utilized data from the 2011–2015 CHARLS database (China Health and Retirement Longitudinal Study, CHARLS), focusing on participants aged 60 years and older. The cross-sectional analysis included 7,744 samples, with 2,398 in the sarcopenia group and 5,346 in the non-sarcopenia group. In the retrospective cohort study, 1,441 participants without sarcopenia at baseline were selected and followed for the development of sarcopenia. Multivariable logistic regression was employed to analyze the association between AIP, BMI, and sarcopenia risk. A restricted cubic spline regression model was used to evaluate the dose-response association, and ROC curve analysis was performed to assess the predictive ability of individual and combined indicators (AIP and BMI). Additionally, subgroup analysis was conducted to explore the association between AIP, BMI, and sarcopenia risk across different demographic groups.

**Results:**

The cross-sectional analysis demonstrated that sarcopenia was significantly associated with various factors, including age, marital status, education level, residence, smoking, BMI, uric acid (UA), glycosylated hemoglobin (HbA1c), total cholesterol (TC), triglycerides (TG), low-density lipoprotein cholesterol (LDL-C), high-density lipoprotein cholesterol (HDL-C), AIP, as well as hypertension, diabetes, dyslipidemia, and heart disease (*p* < 0.05). Logistic regression analysis, adjusted for potential confounders, revealed that the low AIP group was significantly associated with an increased risk of sarcopenia (OR = 1.22, 95% CI 1.03–1.44, *p* = 0.02), while no significant difference was observed in the high AIP group (OR = 0.83, 95% CI 0.69–1.01, *p* = 0.07). In the retrospective cohort study, the low AIP group showed a positive association with sarcopenia risk (OR = 1.79, 95% CI 1.18–2.72, *p* = 0.01), and a similar trend was observed in the high AIP group (OR = 1.69, 95% CI 1.03–2.77, *p* = 0.04). BMI was inversely associated with sarcopenia incidence, consistent with the cross-sectional findings. Both AIP and BMI showed a nonlinear dose-response relationship with sarcopenia risk, with AIP approximating a U-shaped curve and BMI approximating an L-shaped curve. Subgroup analysis indicated that, in the 65–69 age group, low AIP levels were significantly associated with an increased risk of sarcopenia. In participants aged 70 and above, as well as in females, both low and high AIP levels were significantly associated with higher incidence risk. ROC curve analysis showed that the combined use of AIP and BMI for predicting sarcopenia had an Area Under the Curve (AUC) of 0.8913, which was moderately better than the use of AIP (0.6499) or BMI (0.8888) alone.

**Conclusion:**

The changes in AIP and BMI are associated with the risk of sarcopenia, and both provide some predictive value for sarcopenia. Taken together, the combined prediction using AIP and BMI appears to be somewhat more effective than using either indicator alone in assessing the risk of sarcopenia.

**Supplementary Information:**

The online version contains supplementary material available at 10.1007/s40520-025-03029-0.

## Introduction

Sarcopenia is a progressive and systemic skeletal muscle disorder, marked by the accelerated loss of muscle mass and function. This condition can result in adverse outcomes, including falls, functional decline, frailty, and increased mortality. While sarcopenia is prevalent in older adults, middle-aged individuals with multiple underlying diseases are also at higher risk [1]. As defined by the European Working Group on Sarcopenia in Older People (EWGSOP) and the Asian Working Group on Sarcopenia (AWGS), the diagnosis of sarcopenia requires a comprehensive evaluation of muscle mass, muscle strength, and physical performance [2, 3]. The etiology of sarcopenia is multifactorial, including factors such as low physical activity [4], reduced caloric intake, chronic inflammation, oxidative stress, and neurodegenerative processes [5]. Hormonal changes [6, 7], inadequate nutrition [8], and inflammation associated with disease [9, 10] also contribute to the development of sarcopenia. Furthermore, the aging process itself disrupts the metabolic balance of muscle tissue, leading to an imbalance between muscle protein synthesis and degradation, ultimately resulting in skeletal muscle loss [11]. In 2020, the population aged ≥ 60 years in China was 264 million, comprising 18.70% of the total population [12]. As the population continues to age, health issues among older adults are becoming increasingly prominent [13]. The prevalence of sarcopenia among individuals aged ≥ 65 years in China is 17.4% [13], indicating that sarcopenia will pose a significant health challenge for the older population in China.

The atherogenic index of plasma (AIP) is calculated as Log[Triglycerides (TG)/High-Density Lipoprotein Cholesterol (HDL-C)] and is influenced by the size of lipoprotein particles [14]. As a screening tool for dyslipidemia, AIP has garnered increasing attention and is recognized as a major risk indicator for cardiovascular and metabolic diseases [15]. Recent studies have demonstrated a significant positive correlation between AIP and cardiovascular metabolic diseases [16–18]. When AIP values are below 0.11, they are linked to a lower risk of cardiovascular diseases (CVD); values above 0.11 are associated with moderate to high CVD risk [19, 20]. Additionally, AIP has been shown to positively correlate with increases in Body Mass Index (BMI) [21]. BMI, derived from weight and height, is widely used to assess health status and is strongly associated with the risk of various diseases. Studies have indicated that older individuals with low BMI are at higher risk for sarcopenia [22, 23]. Notably, a reciprocal relationship exists between sarcopenia and cardiovascular metabolic diseases: sarcopenia may increase the risk of cardiovascular diseases through insulin resistance and chronic inflammation, while the progression of cardiovascular diseases can exacerbate muscle loss, further promoting sarcopenia. This bidirectional relationship suggests that AIP may not only serve as a tool for predicting cardiovascular disease risk but also provide valuable insights for sarcopenia assessment. However, no studies have yet examined the correlation between AIP and sarcopenia in the Chinese population. This study will, for the first time, explore the relationship between AIP, BMI, and sarcopenia in the older Chinese population through both cross-sectional and retrospective cohort analyses based on the CHARLS database. The goal is to identify high-risk individuals and develop personalized prevention and treatment strategies to prevent sarcopenia.

## Method

### Study population

**Fig. 1:**
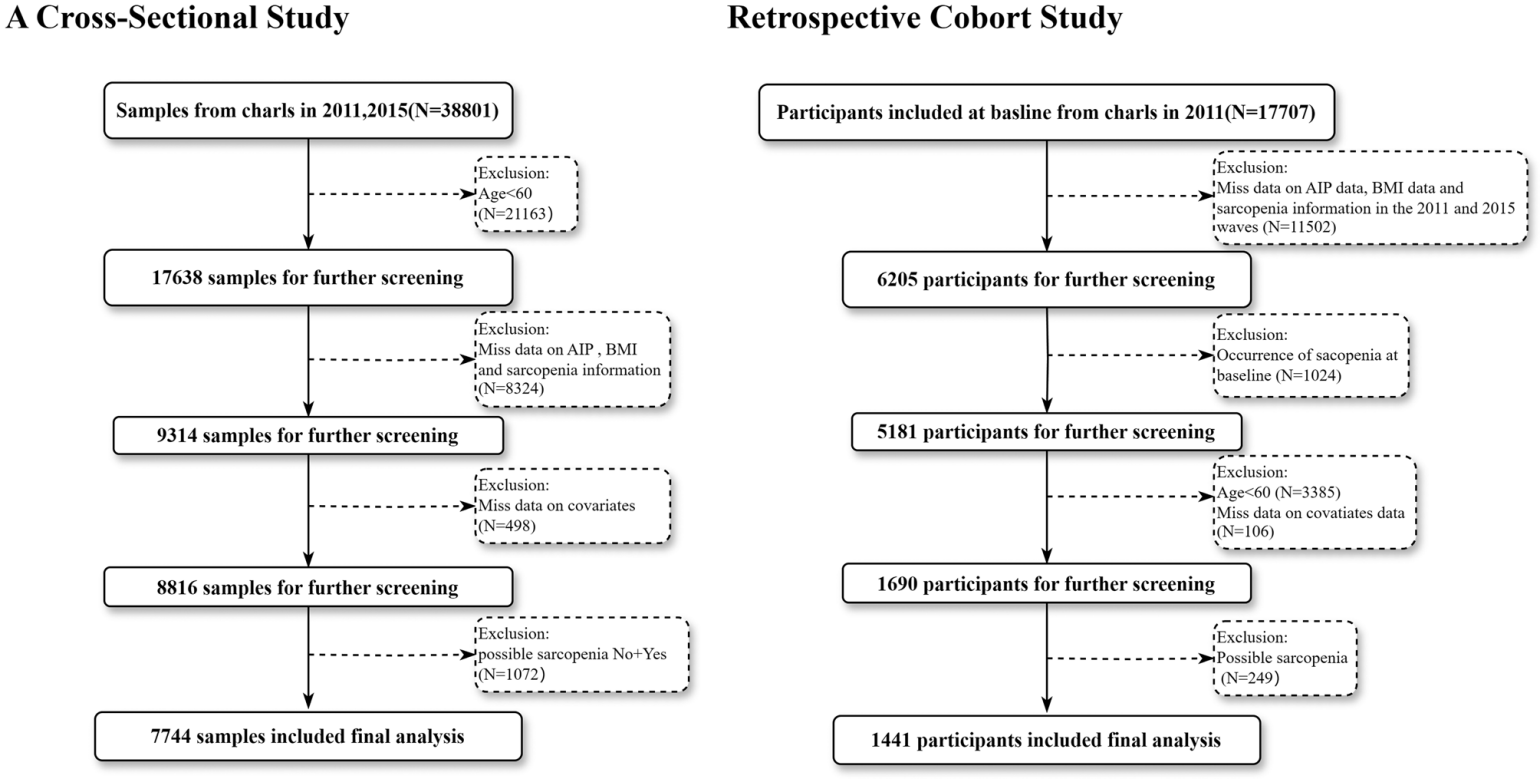
Flowchart of study participant inclusion

The CHARLS database collects high-quality data from individuals aged 45 and above in China to support interdisciplinary research on population aging. In 2011, CHARLS conducted a national baseline survey using a multi-stage probability sampling method, covering approximately 10,000 households across 28 provinces and 150 counties, with over 17,000 respondents participating. Since then, CHARLS has conducted face-to-face interviews with respondents every 2 to 3 years at their residences. The survey covers a wide range of topics, including basic demographic information, household members, health status, healthcare and insurance, employment, income, expenses, and assets. Between 2011 and 2015, researchers collected venous blood samples from respondents who had fasted for at least 12h. These biomedical procedures were carried out by certified professionals following standard operating procedures. Blood samples were stored at 4 °C and promptly sent to the Beijing central laboratory (Capital Medical University, You’anmen Clinical Laboratory) for advanced diagnostic evaluation. Indicators such as blood glucose concentration (GLU), uric acid (UA), glycated hemoglobin (HbA1c), total cholesterol (TC), triglycerides (TG), low-density lipoprotein cholesterol (LDL-C), high-density lipoprotein cholesterol (HDL-C), and C-reactive protein (CRP) were measured using enzyme colorimetric methods [24]. First, we conducted a cross-sectional analysis of the 2011 and 2015 data. Among laboratory test results and sarcopenia-related diagnostic outcomes, we selected the target population with complete records and collected data from eligible participants. After screening, 7,744 samples were included in the analysis, comprising 2,398 sarcopenia samples and 5,346 non-sarcopenia samples. We then designed a retrospective cohort study based on the 2011 data, selecting individuals without sarcopenia at baseline and collecting incident data in 2015. After screening, 1,441 participants were included in the analysis, including 290 sarcopenia patients and 1,151 non-sarcopenia patients. Figure 1 illustrates our research design process. The CHARLS dataset is available for download at the CHARLS official website (http://charls.pku.edu.cn/en). The Biomedical Ethics Review Committee of Peking University approved the collection of CHARLS data (IRB 00001052–11015), and all participants signed an informed consent form.

### Assessment of the AIP

In this study, we used the AIP to assess its potential impact on sarcopenia. The AIP index is calculated using the following formula: AIP = Log(TG/HDL-C) [25], where TG represents triglycerides and HDL-C represents high-density lipoprotein cholesterol. Previous studies [19, 26] have demonstrated that when the AIP index exceeds 0.11, the risk of cardiovascular and metabolic diseases significantly increases. Therefore, individuals with an AIP index greater than 0.11 were classified into the high-AIP group. To further explore the relationship between the AIP index and sarcopenia, we conducted a ROC curve analysis using retrospective cohort data. The analysis identified a best cutoff value of -0.115. Based on this value, we categorized participants into three groups: AIP low-level group (AIP ≤ -0.115), AIP moderate-level group (-0.115 < AIP ≤ 0.11), and AIP high-level group (AIP > 0.11).

### Assessment of the BMI

In this study, BMI was used as an important indicator to assess obesity levels, based on the World Health Organization (WHO) criteria for obesity in Asian populations. According to WHO guidelines [27], BMI is classified into the following four categories: BMI < 18.5: Underweight; 18.5 ≤ BMI < 23: Normal weight; 23 ≤ BMI < 27.5: Overweight; BMI ≥ 27.5: Obesity.

### Assessment of sarcopenia

Grip strength was used to assess muscle strength by measuring the dominant and non-dominant hands, with results recorded in kilograms. Participants were instructed to squeeze the Yuejian™ WL1000 force gauge (Nantong Yuejian Fitness Measurement Instrument Co., Ltd., Nantong, China) as forcefully as possible. The critical values for insufficient grip strength were 28 kg for men and 18 kg for women [28]. Muscle mass was evaluated using Appendicular Skeletal Muscle Mass (ASM), calculated using previously validated anthropometric equations [29]. After calculating ASM, it was adjusted for height by dividing by the square of height (in meters), resulting in height-adjusted muscle mass (ASM/HT²). ASM/HT² values below 5.63 kg/m² for women or 7.05 kg/m² for men were considered indicative of low muscle mass. Physical performance was assessed using a walking speed test and a chair stand test. Detailed evaluation and definition of sarcopenia are available in previous CHARLS studies [29]. In this study, sarcopenia was assessed using the diagnostic algorithm recommended by AWGS 2019 [2]. The diagnostic criteria for sarcopenia include reduced muscle mass along with either reduced muscle strength or low physical performance.

### Assessment of covariates

In this study, baseline data were collected for various covariates, including sociodemographic characteristics, lifestyle behaviors, and current health conditions. Sociodemographic characteristics included age, gender, education level, marital status, and residence (urban or rural); lifestyle behaviors included smoking and alcohol consumption; and current health conditions were recorded as yes/no for hypertension, dyslipidemia, diabetes, chronic lung disease, liver disease, heart disease, and gastrointestinal diseases. Laboratory tests included GLU, UA, HbA1c, TC, TG, LDL-C, HDL-C, and CRP.

### Statistical analysis

Continuous variables were expressed as mean ± standard deviation (SD), and categorical variables as percentages. In this study, AIP levels and BMI were treated as exposure factors to examine their relationship with sarcopenia. Both cross-sectional and retrospective cohort analyses employed multivariable logistic regression to investigate the association between AIP levels, BMI, and sarcopenia risk. Four logistic models were used to calculate the odds ratio (OR) and 95% confidence interval (CI) for sarcopenia risk: Crude Model: the unadjusted crude model; Model 1: adjusted for sociodemographic factors and lifestyle behaviors, including age, gender, education level, location, marital status, smoking, and alcohol consumption; Model 2: adjusted for current health conditions, including (yes/no) hypertension, dyslipidemia, diabetes, chronic lung disease, liver disease, heart disease, and stomach diseases; Model 3: further adjusted for laboratory test indicators, including HbA1c, UA, TC, GLU, CRP. Additionally, in the retrospective cohort analysis, restricted cubic spline (RCS) methods were used to visually depict the dose-response relationship between AIP, BMI, and sarcopenia risk across the four models. Subgroup analysis and interaction assessments were conducted for a more detailed analysis. Finally, the predictive value of individual and combined indicators for sarcopenia was assessed using the receiver operating characteristic (ROC) curve. All statistical analyses were performed using R version 4.4.2 software, with a significance level set at α = 0.05.

## Results

### Results of cross-sectional study

#### Study population characteristics

Table 1 describes the characteristics of the study sample. Among the 7744 samples included, 2398 were diagnosed with sarcopenia. The study sample consisted of 50.15% males and 49.85% females. Comparative analysis showed that, compared to non-sarcopenic patients, those with sarcopenia exhibited the following characteristics: older age, unmarried status, lower education level, rural residence, smoking, and the absence of hypertension, dyslipidemia, heart disease, kidney disease, diabetes, or liver disease. However, they had a higher prevalence of chronic lung disease and stomach disease (*p* < 0.05). Regarding laboratory results, sarcopenic patients showed lower levels of BMI, AIP, HbA1c, TC, UA, LDL-C, and TG, but higher levels of HDL-C.

**Table 1:** Characteristics of cross-sectional study population by sarcopenia

	Total(*n* = 7744)	Sarcopenia (*n* = 2398)	Non-sarcopenia(*n* = 5346)	*p*.value
**Age**				< 0.0001
60–64	3015(38.93)	533(22.23)	2482(46.43)	
65–69	2175(28.09)	625(26.06)	1550(28.99)	
≥ 70	2554(32.98)	1240(51.71)	1314(24.58)	
**Gender**				0.63
Female	3860(49.85)	1185(49.42)	2675(50.04)	
Male	3884(50.15)	1213(50.58)	2671(49.96)	
**Marital status**				< 0.0001
Non-Married	1504(19.42)	630(26.27)	874(16.35)	
Married	6240(80.58)	1768(73.73)	4472(83.65)	
**Education**				< 0.0001
High school or above	490(6.33)	84(3.50)	406(7.59)	
Junior high school or below	4597(59.36)	1281(53.42)	3316(62.03)	
Illiterate	2657(34.31)	1033(43.08)	1624(30.38)	
**Location**				< 0.0001
Rural	4970(64.18)	1803(75.19)	3167(59.24)	
Urban	2774(35.82)	595(24.81)	2179(40.76)	
**Smoke**				< 0.0001
No	5422(70.02)	1560(65.05)	3862(72.24)	
Yes	2322(29.98)	838(34.95)	1484(27.76)	
**Drink**				0.10
No	5135(66.31)	1622(67.64)	3513(65.71)	
Yes	2609(33.69)	776(32.36)	1833(34.29)	
**Hypertension**				< 0.0001
No	5053(65.25)	1830(76.31)	3223(60.29)	
Yes	2691(34.75)	568(23.69)	2123(39.71)	
**Dyslipidemia**				< 0.0001
No	6666(86.08)	2235(93.20)	4431(82.88)	
Yes	1078(13.92)	163(6.80)	915(17.12)	
**Diabetes**				< 0.0001
No	7056(91.12)	2286(95.33)	4770(89.23)	
Yes	688(8.88)	112(4.67)	576(10.77)	
**Chronic lung diseases**				< 0.0001
No	6628(85.59)	1942(80.98)	4686(87.65)	
Yes	1116(14.41)	456(19.02)	660(12.35)	
**Liver disease**				0.23
No	7403(95.60)	2303(96.04)	5100(95.40)	
Yes	341(4.40)	95(3.96)	246(4.60)	
**Heart disease**				< 0.0001
No	6425(82.97)	2064(86.07)	4361(81.58)	
Yes	1319(17.03)	334(13.93)	985(18.42)	
**Kidney disease**				0.02
No	7194(92.90)	2253(93.95)	4941(92.42)	
Yes	550(7.10)	145(6.05)	405(7.58)	
**Stomach disease**				< 0.0001
No	5840(75.41)	1725(71.93)	4115(76.97)	
Yes	1904(24.59)	673(28.07)	1231(23.03)	
**TC(mg/dl)**	189.58 ± 38.10	185.27 ± 39.28	191.51 ± 37.41	< 0.001
**HDL-C(mg/dl)**	52.43 ± 13.79	57.09 ± 14.70	50.35 ± 12.82	< 0.0001
**LDL-C(mg/dl)**	110.07 ± 32.75	105.81 ± 32.34	111.98 ± 32.76	< 0.0001
**TG(mg/dl)**	127.47 ± 82.78	101.92 ± 57.41	138.93 ± 89.58	< 0.0001
**HbA1c(mg/dl)**	5.73 ± 0.97	5.59 ± 0.86	5.79 ± 1.01	< 0.0001
**CRP (mg/dl)**	3.01 ± 7.42	3.28 ± 9.27	2.89 ± 6.42	0.06
**GLU(mg/dl)**	105.02 ± 31.75	100.52 ± 30.98	107.03 ± 31.88	< 0.0001
**UA(mg/dl)**	4.82 ± 1.36	4.57 ± 1.37	4.93 ± 1.35	< 0.0001
**AIP Level**				< 0.0001
Low	3104(40.08)	1392(58.05)	1712(32.02)	
Moderate	2308(29.80)	660(27.52)	1648(30.83)	
High	2332(30.11)	346(14.43)	1986(37.15)	
**BMI(kg/m2)**				< 0.0001
<18.5	764(9.87)	681(28.40)	83(1.55)	
18.5 ~ 23	3347(43.22)	1685(70.27)	1662(31.09)	
23 ~ 27.5	773(9.98)	22(0.92)	751(14.05)	
>27.5	2860(36.93)	10(0.42)	2850(53.31)	

P values were calculated using the Chi-square test (for categorical variables), the Mann-Whitney U test or Wilcoxon rank-sum test (for continuous variables with non-normal distribution), or the Student’s t-test (for continuous variables with normal distribution)

#### Relationship between AIP, BMI and sarcopenia

Sarcopenia was considered the dependent variable (sarcopenia = 1, non-sarcopenia = 0), with BMI and AIP levels as independent variables in the logistic regression analysis. The moderate AIP group was used as the reference group, and the BMI group of 18.5–23 was used as the reference group. The results are shown in Table 2. Regarding the AIP index, in model 3, after full adjustment, the low AIP group had a significantly higher incidence of sarcopenia compared to the moderate AIP group (OR = 1.22, 95% CI 1.03–1.44, *p* = 0.02), whereas the high AIP group showed no significant difference in sarcopenia incidence (OR = 0.83, 95% CI 0.69–1.01, *p* = 0.07). Regarding BMI, all four models demonstrated a significant inverse relationship between BMI and the incidence of sarcopenia. In the 23-27.5 BMI group, the odds ratio for sarcopenia incidence was 0.02 (95% CI 0.01, 0.02) in all four models, representing a 98% reduction in the risk of developing sarcopenia.

**Table 2:** Logistic regression results for AIP, BMI, and the risk of sarcopenia

Character	Crude Model		Model 1		Model 2		Model 3	
	OR(95%CI)	*P*	OR(95%CI)	*P*	OR(95%CI)	*P*	OR(95%CI)	*P*
**Sarcopenia ~ AIP_Level**								
Moderate	ref		ref		ref		ref	
Low	2.03(1.81,2.28)	< 0.0001	2.11(1.87,2.39)	< 0.0001	1.98(1.75,2.25)	< 0.0001	1.22(1.03,1.44)	0.02
High	0.44(0.38,0.50)	< 0.0001	0.47(0.40,0.55)	< 0.0001	0.51(0.44,0.60)	< 0.0001	0.83(0.69,1.01)	0.07
p for trend		< 0.0001		< 0.0001		< 0.0001		0.4
**Sarcopenia ~ BMI**								
18.5 ~ 23	ref		ref		ref		ref	
<18.5	9.94(8.06,12.26)	< 0.0001	9.56(7.70,11.87)	< 0.0001	9.35(7.52,11.63)	< 0.0001	8.79(7.05,10.94)	< 0.0001
23 ~ 27.5	0.02(0.01,0.02)	< 0.0001	0.02(0.01,0.02)	< 0.0001	0.02(0.01,0.02)	< 0.0001	0.02(0.01,0.02)	< 0.0001
>27.5	0.01(0.00,0.01)	< 0.0001	0.01(0.00,0.01)	< 0.0001	0.01(0.00,0.01))	< 0.0001	0.01(0.00,0.01)	< 0.0001
p for trend		< 0.0001		< 0.0001		< 0.0001		< 0.0001

**Crude Model**: Unadjusted

**Model 1**: Adjusted for age, sex, education level, location, marital status, smoking, and drinking status

**Model 2**: Additionally adjusted for the presence of hypertension, dyslipidemia, diabetes, chronic lung disease, liver disease, heart disease, kidney disease, and stomach disease

**Model 3**: For sarcopenia and AIP, Model 3 further adjusted for HbA1c, UA, TC, GLU, CRP, and BMI; for sarcopenia and BMI, Model 3 further adjusted for HbA1c, UA, TC, GLU, CRP, and AIP

### Retrospective cohort study results

#### Study population characteristics

Table 3 presents the baseline characteristics of the study participants. Of the final 1441 participants, 290 were diagnosed with sarcopenia. The study population was composed of 50.73% males and 49.27% females. Comparative analysis revealed that, compared to non-sarcopenia participants, those with sarcopenia were older, more likely to be non-married, reside in rural areas, and smoke, while less likely to have hypertension, dyslipidemia, or diabetes (*p* < 0.05). Laboratory results showed that sarcopenia participants had lower levels of BMI, AIP, GLU, HbA1c, LDL-C, and TG, but higher HDL-C levels.

We also described the baseline characteristics by AIP levels and BMI groups (Tables S1 and S2). AIP levels were divided into three groups (Low: *N* = 530, Moderate: *N* = 404, High: *N* = 507). Significant differences (*p* < 0.05) were observed across groups for several baseline characteristics, including gender, residence, drink, hypertension, dyslipidemia, diabetes, TC, HDL-C, LDL-C, TG, HbA1c, GLU, UA, and BMI. The proportion of females increased with higher AIP levels (Low: 43.21%, High: 54.64%, *p* < 0.001), while the proportion of rural residents decreased (Low: 70.75%, High: 57.59%, *p* < 0.0001). Other variables such as age, marital status, education level, smoking, chronic lung disease, liver disease, heart disease, kidney disease, stomach diseases, and CRP did not show significant differences between the groups.

Similarly, several variables differed significantly between the BMI groups, including gender, age, marital status, residence, smoking, drink, hypertension, dyslipidemia, diabetes, heart disease, TC, HDL-C, LDL-C, TG, HbA1c, and UA (*p* < 0.05). The proportion of females significantly increased with higher BMI (Low weight: 47.83%, Obesity: 57.86%, *p* < 0.0001), while the proportion of rural residents decreased (Low weight: 86.96%, Obesity: 54.72%, *p* < 0.0001). Other variables such as education level, chronic lung disease, liver disease, kidney disease, stomach diseases, and CRP did not show significant differences between the groups.

**Table 3:** Baseline characteristics of the retrospective cohort study population by sarcopenia

	Total(*n* = 1441)	Sarcopenia (*n* = 290)	Non-sarcopenia(*n* = 1151)	*p*.value
**Age**				< 0.0001
60–64	757(52.53)	120(41.38)	637(55.34)	
65–69	397(27.55)	76(26.21)	321(27.89)	
≥ 70	287(19.92)	94(32.41)	193(16.77)	
**Gender**				0.27
Female	710(49.27)	134(46.21)	576(50.04)	
Male	731(50.73)	156(53.79)	575(49.96)	
**Marital status**				< 0.0001
Non-Married	215(14.92)	58(20.00)	157(13.64)	
Married	1226(85.08)	232(80.00)	994(86.36)	
**Education**				0.17
High school or above	88(6.11)	12(4.14)	76(6.60)	
Illiterate	428(29.70)	95(32.76)	333(28.93)	
Junior high school or below	925(64.19)	183(63.10)	742(64.47)	
**Location**				< 0.0001
Rural	917(63.64)	229(78.97)	688(59.77)	
Urban	524(36.36)	61(21.03)	463(40.23)	
**Smoke**				< 0.0001
No	1001(69.47)	175(60.34)	826(71.76)	
Yes	440(30.53)	115(39.66)	325(28.24)	
**Drink**				0.43
No	950(65.93)	185(63.79)	765(66.46)	
Yes	491(34.07)	105(36.21)	386(33.54)	
**Hypertension**				< 0.0001
No	948(65.79)	234(80.69)	714(62.03)	
Yes	493(34.21)	56(19.31)	437(37.97)	
**Dyslipidemia**				< 0.0001
No	1264(87.72)	280(96.55)	984(85.49)	
Yes	177(12.28)	10(3.45)	167(14.51)	
**Diabetes**				< 0.01
No	1334(92.57)	281(96.90)	1053(91.49)	
Yes	107(7.43)	9(3.10)	98(8.51)	
**Chronic lung diseases**				0.79
No	1271(88.20)	254(87.59)	1017(88.36)	
Yes	170(11.80)	36(12.41)	134(11.64)	
**Liver disease**				0.67
No	1393(96.67)	282(97.24)	1111(96.52)	
Yes	48(3.33)	8(2.76)	40(3.48)	
**Heart disease**				0.91
No	1223(84.87)	245(84.48)	978(84.97)	
Yes	218(15.13)	45(15.52)	173(15.03)	
**Kidney disease**				0.95
No	1358(94.24)	274(94.48)	1084(94.18)	
Yes	83(5.76)	16(5.52)	67(5.82)	
**Stomach disease**				0.41
No	1112(77.17)	218(75.17)	894(77.67)	
Yes	329(22.83)	72(24.83)	257(22.33)	
**TC(mg/dl)**	197.07 ± 38.23	192.95 ± 38.31	198.11 ± 38.16	0.04
**HDL-C(mg/dl)**	50.35 ± 14.71	56.44 ± 16.85	48.82 ± 13.71	< 0.0001
**LDL-C(mg/dl)**	120.61 ± 35.21	115.59 ± 35.05	121.88 ± 35.15	< 0.01
**TG(mg/dl)**	131.50 ± 95.98	106.99 ± 68.51	137.68 ± 100.83	< 0.0001
**HbA1c(mg/dl)**	5.34 ± 0.75	5.22 ± 0.55	5.37 ± 0.78	< 0.001
**CRP(mg/dl)**	2.76 ± 6.05	2.45 ± 5.17	2.84 ± 6.25	0.28
**GLU(mg/dl)**	110.67 ± 30.80	106.27 ± 24.10	111.77 ± 32.19	< 0.01
**UA(mg/dl)**	4.63 ± 1.25	4.49 ± 1.26	4.67 ± 1.24	0.03
**AIP**	0.00 ± 0.32	-0.12 ± 0.32	0.04 ± 0.32	< 0.0001
**AIP Level**				< 0.0001
Low	530(36.78)	166(57.24)	364(31.62)	
Moderate	404(28.04)	61(21.03)	343(29.80)	
High	507(35.18)	63(21.72)	444(38.58)	
**BMI(kg/m2)**				< 0.0001
<18.5	46(3.19)	41(14.14)	5(0.43)	
18.5 ~ 23	532(36.92)	218(75.17)	314(27.28)	
23 ~ 27.5	545(37.82)	26(8.97)	519(45.09)	
>27.5	318(22.07)	5(1.72)	313(27.19)	

#### Relationship between AIP, BMI and sarcopenia

Logistic regression analysis was performed with sarcopenia (1 = sarcopenia, 0 = non-sarcopenia) as the dependent variable and AIP and BMI as independent variables. The moderate AIP group and the BMI group of 18.5–23 served as reference groups. The results are shown in Table 4.

In the crude model, the low AIP group had a significantly higher risk of sarcopenia compared to the reference group (OR: 2.56, 95% CI: 1.85–3.56, *p* < 0.0001). This trend remained significant in the adjusted models (Model 1–3) (Model 3: OR: 1.79, 95% CI: 1.18–2.72, *p* = 0.01). Interestingly, in the fully adjusted Model 3, the high AIP group showed a significant positive association with the reference group (OR: 1.69, 95% CI: 1.03–2.77, *p* = 0.04), suggesting that high AIP may be associated with sarcopenia, especially after fully adjusting for potential confounders.

Regarding BMI, all BMI groups showed significant differences compared to the reference group (18.5–23). In all models, the OR for the BMI < 18.5 group was significantly elevated, with p-values < 0.0001, indicating a strong positive correlation between low BMI and sarcopenia. For the BMI groups of 23-27.5 and > 27.5, the OR values were significantly below 1 in all models, with p-values < 0.0001, suggesting that higher BMI may be linked to a reduced risk of sarcopenia.

**Table 4:** Logistic regression results for AIP, BMI, and the risk of sarcopenia

Character	Crude Model		Model 1		Model 2		Model 3	
	OR(95%CI)	*P*	OR(95%CI)	*P*	OR(95%CI)	*P*	OR(95%CI)	*P*
**Sarcopenia ~ AIP_Level**								
Moderate	ref		ref		ref		ref	
Low	2.56(1.85,3.56)	< 0.0001	2.44(1.74,3.43)	< 0.0001	2.34(1.65,3.31)	< 0.0001	1.79(1.18,2.72)	0.01
High	0.8(0.55,1.17)	0.24	0.84(0.57,1.24)	0.37	0.92(0.62,1.36)	0.67	1.69(1.03,2.77)	0.04
p for trend		0.12		0.23		0.55		0.03
**Sarcopenia ~ BMI**								
18.5 ~ 23	ref		ref		ref		ref	
<18.5	11.81(4.59,30.37)	< 0.0001	10.27(3.92,26.94)	< 0.0001	10.85(4.12,28.54)	< 0.0001	10.84(4.09,28.71)	< 0.0001
23 ~ 27.5	0.07(0.05, 0.11)	< 0.0001	0.07(0.05, 0.11)	< 0.0001	0.08(0.05, 0.12)	< 0.0001	0.08(0.05, 0.13)	< 0.0001
>27.5	0.02(0.01, 0.06)	< 0.0001	0.02(0.01, 0.06)	< 0.0001	0.03(0.01, 0.07)	< 0.0001	0.03(0.01, 0.08)	< 0.0001
p for trend		< 0.0001		< 0.0001		< 0.0001		< 0.0001

* **Crude Model**: Unadjusted

**Model 1**: Adjusted for age, sex, education level, location, marital status, smoking, and drinking status

**Model 2**: Additionally adjusted for the presence of hypertension, dyslipidemia, diabetes, chronic lung disease, liver disease, heart disease, kidney disease, and stomach disease

**Model 3**: For sarcopenia and AIP, Model 3 further adjusted for HbA1c, UA, TC, GLU, CRP, and BMI; for sarcopenia and BMI, Model 3 further adjusted for HbA1c, UA, TC, GLU, CRP, and AIP

#### Dose-response relationship of AIP and BMI with sarcopenia

**Fig. 2:**
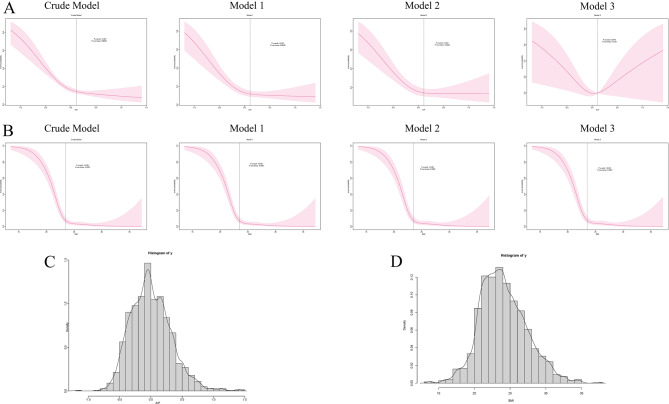
Association between AIP (**A**) and BMI (**B**) and probability of sarcopenia in restricted cubic spline analysis

Figure 2 presents the results of the RCS analysis. The x-axis depicts the continuous variation of different indicators, while the y-axis shows the event probability. Parts C and D illustrate the data distribution. In the fully adjusted model, a significant non-linear dose-response relationship was observed between AIP, BMI, and sarcopenia risk (AIP: P overall = 0.0476, P non-linear = 0.019; BMI: P overall < 0.001, P non-linear = 0.0007). In Model 3, after full adjustment, the cut-off between low and high AIP levels was set at 0.11. When AIP is below 0.11, the probability of sarcopenia decreases as AIP rises. However, when AIP exceeds 0.11, the probability of sarcopenia may increase with AIP.

The relationship between BMI and sarcopenia risk follows an “L-shaped” pattern. The cut-off between normal weight and overweight was set at a BMI of 23. When BMI is below 23, an increase in BMI is significantly negatively correlated with sarcopenia risk, with a lower probability of sarcopenia as BMI increases. However, when BMI exceeds 23, the effect of BMI on sarcopenia probability weakens significantly.

#### Stratified analysis

Subgroup analysis was conducted by adjusting for hypertension, dyslipidemia, diabetes, chronic lung disease, liver disease, heart disease, stomach diseases, AIP levels and BMI, as well as all other categorical variables, which were incorporated into the binary regression model for subgroup analysis (Tables 5 and 6).

The effects of AIP levels on sarcopenia varied across different age and sex strata. In the 60–64 age group, AIP levels did not appear to be significantly associated with the occurrence of sarcopenia. In the 65–69 age group, the low AIP group had a significantly higher risk of sarcopenia compared to the moderate AIP group, while the high AIP group did not show significant differences. Interestingly, among those aged 70 and above, both low and high AIP levels were significantly associated with an increased risk of sarcopenia compared to moderate AIP levels. This aligns with the results from previous regression and RCS analyses. This trend was also significant in the female subgroup. The BMI subgroup analysis showed that, across all age and sex strata, the risk of sarcopenia decreased progressively as BMI increased from the underweight group to the overweight group. However, the obesity group did not show a significant effect on sarcopenia risk compared to the overweight group.

**Table 5:** Stratified analysis of the effect of AIP levels on sarcopenia by age and gender

	Moderate	Low	*P*	High	*P*	*p* for interaction
**Age**						0.102
60–64	ref	1.179(0.653,2.155)	0.588	0.825(0.404,1.670)	0.593	
65–69	ref	2.381(1.134,5.148)	0.024	1.711(0.715,4.161)	0.230	
≥70	ref	4.486(1.885,11.447)	0.001	3.619(1.404, 9.901)	0.009	
**Gender**						0.264
Male	ref	1.442(0.854,2.464)	0.174	1.031(0.525,2.014)	0.928	
Female	ref	2.453(1.361,4.524)	0.003	1.925(1.025,3.689)	0.044	

Adjusted for the presence of hypertension, dyslipidemia, diabetes, chronic lung disease, liver disease, heart disease, stomach diseases, and BMI

**Table 6:** Stratified analysis of the effect of BMI on sarcopenia by age and gender

	23 ~ 27.5	<18.5	*p*	18.5 ~ 23	*p*	>27.5	*p*	*p* for interaction
**Age**								0.588
60–64	ref	208.476(45.702,1571.597)	< 0.0001	15.129(7.489,34.951)	< 0.0001	0.252(0.013,1.410)	0.197	
65–69	ref	59.059(10.042,513.474)	< 0.0001	15.127(6.677,39.521)	< 0.0001	0.321(0.017,1.904)	0.296	
≥ 70	ref	165.052(27.769,3217.717)	< 0.0001	9.903(4.528,23.528)	< 0.0001	0.461(0.095,1.682)	0.276	
**Gender**								0.082
Male	ref	662.806(112.677,12898.518)	< 0.0001	18.794(8.996,46.035)	< 0.0001	0.302(0.016,1.751)	0.268	
Female	ref	58.718(17.928,238.098)	< 0.0001	9.904(5.784,17.739)	< 0.0001	0.355(0.100,0.982)	0.068	

Adjusted for the presence of hypertension, dyslipidemia, diabetes, chronic lung disease, liver disease, heart disease, stomach diseases, and AIP Level

#### Predictive value of AIP index and BMI for sarcopenia

**Fig. 3:**
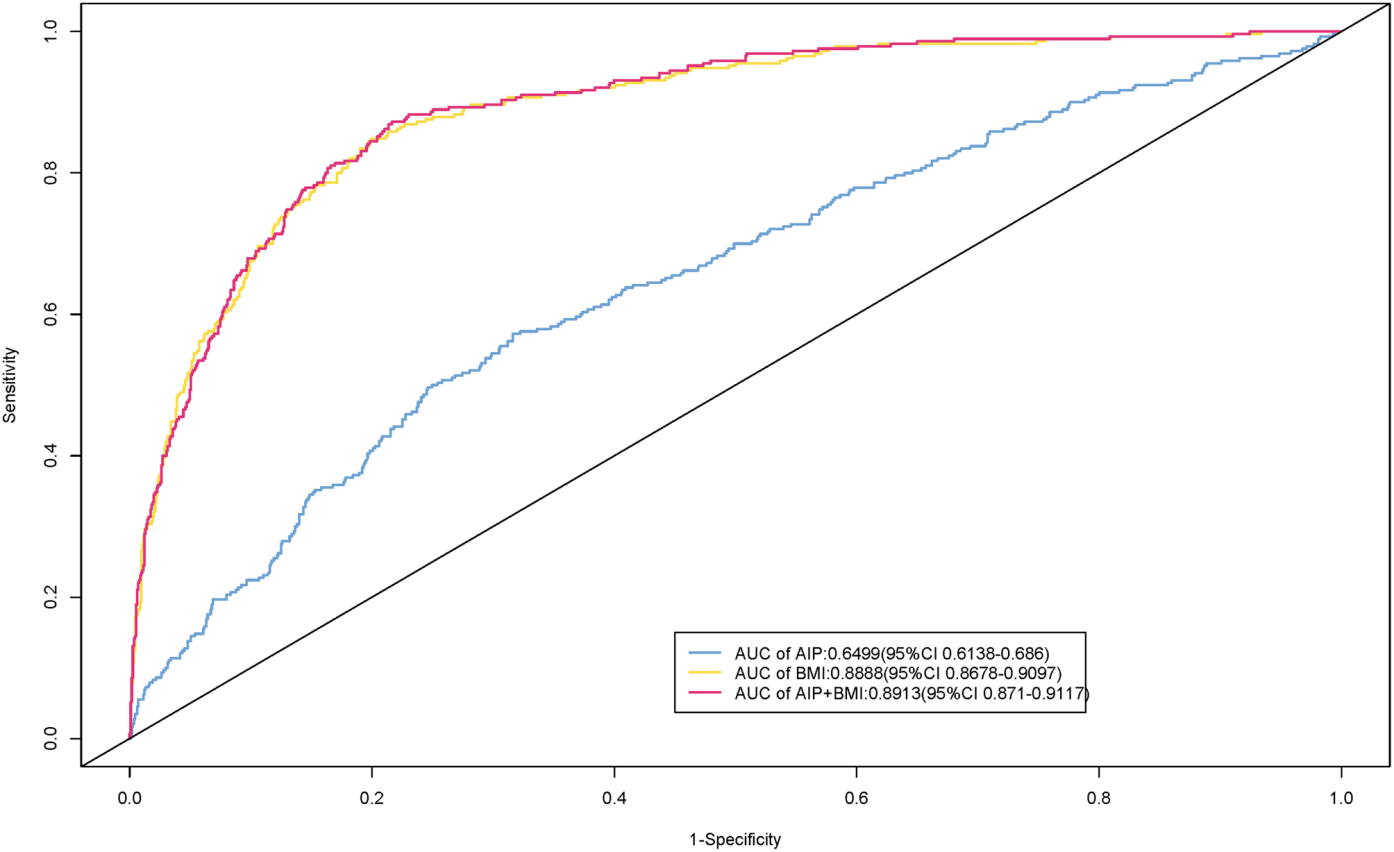
ROC curves for predicting sarcopenia occurrence using the AIP, BMI, and their combined metrics

We used generalized linear models to examine the relationship between AIP, BMI, and their combination with sarcopenia, and employed RCS to capture the nonlinear relationships between AIP, BMI, and sarcopenia. Finally, ROC curves were plotted for each model to evaluate the predictive ability of the different indicators, as shown in the Fig. 3. The AUC values for predicting sarcopenia were as follows: AIP index (0.6499, 95% CI 0.6138–0.686), BMI (0.8888, 95% CI 0.8678–0.9097), and the combination of AIP and BMI (0.8913, 95% CI 0.871–0.911). The combination of AIP and BMI showed the best predictive performance.

## Discussion

Many studies have shown that the AIP can serve as a sensitive biomarker for various diseases. In identifying high-risk populations for diabetes, a cross-sectional study based on CHARLS found a nonlinear relationship between AIP and the risk of type 2 diabetes (T2D). When AIP > -0.04, AIP was positively correlated with the risk of T2D [30]. This finding is consistent with our study, which shows that as AIP levels increase, the risk of diabetes significantly increases (Table S1). Additionally, univariate analysis results also indicate a significant correlation between diabetes and sarcopenia (Tables 1 and 2). There is an inherent physiological and pathological link between diabetes and sarcopenia—insulin resistance. Insulin resistance refers to a complex state in which the sensitivity and responsiveness of insulin target organs or tissues (such as skeletal muscle, liver, and adipose tissue) to endogenous or exogenous insulin are reduced, resulting in decreased efficiency of glucose uptake and utilization compared to normal levels [31]. Insulin resistance mainly causes muscle weakness through two mechanisms [32]: firstly, insulin resistance blocks the signaling of the phosphoinositide 3-kinase (PI3K)/protein kinase B (Akt) pathway, inhibiting protein synthesis and leading to a reduction in muscle proteins and muscle atrophy. Secondly, it inhibits the movement of glucose transporter 4 (GLUT4) from storage vesicles to the plasma membrane, reducing muscle glycogen synthesis and resulting in insufficient energy supply to muscles. Furthermore, insulin resistance induces autophagy and mitochondrial dysfunction, further weakening muscle strength. Hyperglycemia, one of the main characteristics of diabetes, leads to the accumulation of advanced glycation end products (AGEs) in muscles over time. Increased AGEs are closely associated with a decline in muscle mass and strength [33]. Diabetic neuropathy, a common complication in diabetic patients, may lead to rapid degeneration of axons in motor neurons, affecting normal muscle function and causing muscle atrophy [34]. Additionally, neuropathy may impair the sensory feedback system, affecting the body’s balance control ability, and eventually lead to a decline in physical function [35]. In identifying high-risk populations for coronary heart disease, AIP levels can effectively assess risk. Studies show that elevated AIP levels are significantly associated with the risk of coronary heart disease, aiding in the early detection of potential coronary heart disease risk [36]. As shown in Table S1, with the increase in AIP levels, the number of individuals with heart disease significantly increases (*p* < 0.001). Sarcopenia and coronary artery disease share similar underlying biological mechanisms, namely mild chronic systemic inflammation. Therefore, sarcopenia is more common in adults with coronary artery disease [37]. CTRP9 primarily exerts anti-diabetic and glucose-lowering effects by improving insulin sensitivity. Research has found that serum CTRP9 levels are significantly lower in patients with coronary heart disease, and insulin resistance significantly promotes the occurrence and progression of sarcopenia [38]. In identifying metabolic syndrome, a 9-year longitudinal study showed a significant association between AIP levels and metabolic syndrome. Metabolic syndrome is a condition involving multiple metabolic abnormalities and is considered an important risk factor for cardiovascular and cerebrovascular diseases [39]. The characteristics of this syndrome include central obesity, elevated blood glucose levels, insulin resistance, dyslipidemia, and hypertension. The association between metabolic syndrome and sarcopenia may be similar to the mechanisms observed in diabetes, primarily promoting insulin resistance and exacerbating inflammatory responses, thereby increasing the risk of sarcopenia [40].

However, it is important to note that low AIP levels may also have a potential impact on sarcopenia in certain situations. Although high AIP levels are associated with various metabolic diseases such as diabetes, coronary heart disease, and metabolic syndrome, low AIP levels should not be overlooked, especially in the elderly population. Low AIP levels may indirectly reflect abnormal lipid metabolism and low BMI [21, 41, 42], leading to muscle loss and the development of sarcopenia. Previous studies have shown that individuals with low BMI have a higher risk of sarcopenia [43, 44]. Low AIP values reflect a reduced level of fatty acid metabolism, which is often associated with low body weight or malnutrition [45]. Low body weight and insufficient fat reserves often lead to a lack of muscle support, thereby increasing the risk of sarcopenia.

In this study, we explored the relationship between AIP levels and sarcopenia risk using both cross-sectional and retrospective studies. The results show that the impact of different AIP levels on sarcopenia significantly varies and may be influenced by an individual’s age, sex, and other confounding factors. In the cross-sectional study, after adjusting for confounding factors, the logistic regression analysis indicated that low AIP levels were associated with a higher risk of sarcopenia (OR = 1.22, 95% CI: 1.03–1.44, *P* = 0.02). This suggests that in individuals with low AIP levels, the risk of sarcopenia may increase. However, the risk in the high AIP group did not show significant changes (OR = 0.83, 95% CI: 0.69–1.01, *P* = 0.07). The retrospective study further validated this finding, particularly after adjusting for confounding factors. The low AIP group showed a significantly increased risk of sarcopenia (OR = 1.79, 95% CI: 1.18–2.72, *P* = 0.01), consistent with the cross-sectional study. This suggests that low AIP levels may be an independent risk factor for sarcopenia. However, the high AIP group also exhibited a certain increase in risk in the retrospective study (OR = 1.69, 95% CI: 1.03–2.77, *P* = 0.04), with potential mechanisms related to the previously mentioned factors. The RCS analysis suggested that the relationship between AIP and sarcopenia risk may follow a U-shaped curve. When AIP values were below 0.11, an increase in AIP levels was associated with a reduced risk of sarcopenia; however, when AIP values were above 0.11, the risk of sarcopenia increased with higher AIP levels. This U-shaped relationship may reflect the connection between low AIP levels and low BMI or insufficient lipid metabolism, whereas high AIP levels could be associated with a more severe metabolic burden (such as insulin resistance or hyperglycemia), thus increasing the risk of sarcopenia.

Further retrospective cohort stratification studies showed that the relationship between AIP levels and sarcopenia risk exhibited heterogeneity across different age groups and sex. The risk in the low AIP group significantly increased in the population aged ≥ 65 years, with a higher risk observed in women. In the high AIP group, sarcopenia risk also increased in the population aged ≥ 70 years, particularly in older women. This finding emphasizes the need for more attention to monitoring muscle mass and early intervention in older patients with high AIP levels, especially women, in clinical practice, to reduce the health risks associated with sarcopenia.

In many previous studies, higher BMI has been closely associated with lipid metabolism in the older adults and has been identified as a risk factor for various diseases [46]. However, some scholars have proposed the “obesity paradox,” suggesting that obesity may lead to better prognoses [47]. The results of this study also support this viewpoint, showing that as BMI increases, the risk of sarcopenia significantly decreases. However, the relationship between BMI and sarcopenia risk may not be linear and is more likely to follow an “L-shaped” association. In the RCS regression model for BMI (as shown in Fig. 2B), when BMI < 23, the risk of sarcopenia significantly decreased as BMI increased. However, when BMI > 23, although the risk of sarcopenia continued to decrease, the rate of decline was less pronounced compared to when BMI was lower. This further suggests the existence of an “L-shaped” association between BMI and sarcopenia. Additionally, subgroup analysis of BMI showed that when the overweight group was used as the reference, the risk of sarcopenia significantly decreased as BMI increased when BMI was low. However, the risk of sarcopenia in the obese group was not significantly different from that in the overweight group. This suggests that within the normal BMI range, maintaining a higher BMI may help reduce the risk of sarcopenia, while excessive obesity may not provide additional protective effects.

From the above research, it is evident that there is a close relationship between AIP index, BMI levels, and sarcopenia. By plotting the ROC curve, the AUCs for predicting sarcopenia using AIP, BMI, and the combined AIP + BMI were 0.6499, 0.8888, and 0.8913, respectively, all demonstrating strong predictive power. The combined prediction using AIP + BMI was moderately better than using either indicator alone. Given the simplicity of obtaining AIP and BMI values, these indicators can provide effective reference for older individuals in China, facilitating early screening for sarcopenia.

## Limitation

In this study, we employed both cross-sectional and retrospective cohort analyses to explore the relationship between AIP and BMI levels and sarcopenia. Although the study provides valuable insights, there are several limitations, particularly concerning missing data. We used data completeness as a selection criterion for the study population, which helped ensure the quality of the data used in the analysis, reduce bias due to missing data, and improve the accuracy and reliability of the results. However, with a large number of missing values across multiple variables, the sample size was significantly reduced, which could result in a decrease in statistical power and affect the external validity of the findings. Therefore, future research should strengthen the control of missing data during the data collection phase to ensure data completeness and representativeness. Additionally, as this study relied on existing cross-sectional and cohort data, potential confounding factors could not be fully excluded. Despite multiple adjustments, residual confounding may still influence the accuracy of the inferences. As such, while this study provides associations between AIP, BMI, and sarcopenia, it cannot definitively establish causal relationships. In conclusion, while this study offers new insights into the role of AIP and BMI in sarcopenia, considering the limitations of the sample data and design, future studies should involve larger, more comprehensive datasets. Moreover, further prospective, randomized, double-blind studies are recommended to minimize bias and establish clearer causal links, as well as to explore the underlying mechanisms of these associations.

## Conclusion

This study used cross-sectional and retrospective cohort analyses, employing various methods to comprehensively investigate the potential associations between AIP index, BMI levels, and sarcopenia. In populations with low AIP and low BMI, the risk of sarcopenia was significantly increased. Additionally, for females and for individuals aged over 70, excessively high AIP levels might also be associated with an increased risk of sarcopenia, suggesting that AIP monitoring should be emphasized in these groups. Therefore, early intervention targeting these high-risk populations has important clinical significance. Notably, the combined use of AIP and BMI to predict sarcopenia appears to outperform single indicators, highlighting the potential of AIP and BMI as biomarkers for predicting sarcopenia. Given the findings, future research should involve larger sample sizes and explore the underlying mechanisms between AIP, BMI, and sarcopenia to further validate these results and their clinical applicability. Moreover, prospective randomized controlled trials are recommended to confirm the causal relationships and to provide a stronger foundation for clinical application.

## Electronic supplementary material

Below is the link to the electronic supplementary material.

Supplementary Material 1

Supplementary Material 2

## Data Availability

Data is provided within the manuscript or supplementary information files.
