# Supplementary material for: Association between atherogenic index of plasma, body mass index, and sarcopenia: a cross-sectional and longitudinal analysis study based on older adults in China
Source: Aging Clin Exp Res. 2025 Apr 7;37(1):122. doi: 10.1007/s40520-025-03029-0 (PMC11976801; doi:10.1007/s40520-025-03029-0)
Supplement: Supplementary file 1 — Supplementary Material 1 [file 40520_2025_3029_MOESM1_ESM.docx]

**Table S1 Baseline characteristics of study population by AIP**

|  | **Total(n=1441)** | **Low(n=530)** | **Moderate(n=404)** | **High(n=507)** | **p.value** |
| --- | --- | --- | --- | --- | --- |
| **Age** |  |  |  |  | 0.27 |
| 60-64 | 757(52.53) | 277(52.26) | 215(53.22) | 265(52.27) |  |
| 65-69 | 397(27.55) | 133(25.09) | 114(28.22) | 150(29.59) |  |
| ≥70 | 287(19.92) | 120(22.64) | 75(18.56) | 92(18.15) |  |
| **Gender** |  |  |  |  | <0.001 |
| Female | 710(49.27) | 229(43.21) | 204(50.50) | 277(54.64) |  |
| Male | 731(50.73) | 301(56.79) | 200(49.50) | 230(45.36) |  |
| **Marital status** |  |  |  |  | 0.83 |
| Non-Married | 1226(85.08) | 453(85.47) | 340(84.16) | 433(85.40) |  |
| Married | 215(14.92) | 77(14.53) | 64(15.84) | 74(14.60) |  |
| **Education** |  |  |  |  | 0.90 |
| High school or above | 88( 6.11) | 32( 6.04) | 24( 5.94) | 32( 6.31) |  |
| Illiterate | 428(29.70) | 154(29.06) | 128(31.68) | 146(28.80) |  |
| Junior high school or below | 925(64.19) | 344(64.91) | 252(62.38) | 329(64.89) |  |
| **Location** |  |  |  |  | <0.0001 |
| Rural | 917(63.64) | 375(70.75) | 250(61.88) | 292(57.59) |  |
| Urban | 524(36.36) | 155(29.25) | 154(38.12) | 215(42.41) |  |
| **Smoke** |  |  |  |  | 0.10 |
| No | 1001(69.47) | 350(66.04) | 287(71.04) | 364(71.79) |  |
| Yes | 440(30.53) | 180(33.96) | 117(28.96) | 143(28.21) |  |
| **Drink** |  |  |  |  | <0.01 |
| No | 950(65.93) | 319(60.19) | 284(70.30) | 347(68.44) |  |
| Yes | 491(34.07) | 211(39.81) | 120(29.70) | 160(31.56) |  |
| **Hypertension** |  |  |  |  | <0.0001 |
| No | 948(65.79) | 397(74.91) | 257(63.61) | 294(57.99) |  |
| Yes | 493(34.21) | 133(25.09) | 147(36.39) | 213(42.01) |  |
| **Dyslipidemia** |  |  |  |  | <0.0001 |
| No | 1264(87.72) | 495(93.40) | 366(90.59) | 403(79.49) |  |
| Yes | 177(12.28) | 35( 6.60) | 38( 9.41) | 104(20.51) |  |
| **Diabetes** |  |  |  |  | <0.0001 |
| No | 1334(92.57) | 509(96.04) | 375(92.82) | 450(88.76) |  |
| Yes | 107( 7.43) | 21( 3.96) | 29( 7.18) | 57(11.24) |  |
| **Chronic lung diseases** |  |  |  |  | 0.35 |
| No | 1271(88.20) | 459(86.60) | 359(88.86) | 453(89.35) |  |
| Yes | 170(11.80) | 71(13.40) | 45(11.14) | 54(10.65) |  |
| **Liver disease** |  |  |  |  | 0.36 |
| No | 1393(96.67) | 517(97.55) | 389(96.29) | 487(96.06) |  |
| Yes | 48( 3.33) | 13( 2.45) | 15( 3.71) | 20( 3.94) |  |
| **Heart disease** |  |  |  |  | 0.19 |
| No | 1223(84.87) | 459(86.60) | 345(85.40) | 419(82.64) |  |
| Yes | 218(15.13) | 71(13.40) | 59(14.60) | 88(17.36) |  |
| **Kidney disease** |  |  |  |  | 0.14 |
| No | 1358(94.24) | 493(93.02) | 388(96.04) | 477(94.08) |  |
| Yes | 83( 5.76) | 37( 6.98) | 16( 3.96) | 30( 5.92) |  |
| **Stomach disease** |  |  |  |  | 0.80 |
| No | 1112(77.17) | 411(77.55) | 307(75.99) | 394(77.71) |  |
| Yes | 329(22.83) | 119(22.45) | 97(24.01) | 113(22.29) |  |
| **TC(mg/dl)** | 197.07 ± 38.23 | 190.83 ± 34.85 | 195.52 ± 38.32 | 204.84 ± 40.22 | <0.0001 |
| **HDL-C(mg/dl)** | 50.35 ± 14.71 | 62.63 ± 13.34 | 48.89 ± 8.55 | 38.68 ± 8.68 | <0.0001 |
| **LDL-C(mg/dl)** | 120.61 ± 35.21 | 116.85 ± 30.60 | 126.27 ± 34.14 | 120.05 ± 39.75 | <0.001 |
| **TG(mg/dl)** | 131.50 ± 95.98 | 70.47 ± 17.99 | 109.69 ± 21.92 | 212.68 ± 120.87 | <0.0001 |
| **HbA1c(mg/dl)** | 5.34 ± 0.75 | 5.22 ± 0.53 | 5.33 ± 0.81 | 5.47 ± 0.86 | <0.0001 |
| **CRP (mg/dl)** | 2.76 ± 6.05 | 2.55 ± 6.91 | 2.93 ± 6.15 | 2.84 ± 4.89 | 0.59 |
| **GLU(mg/dl)** | 110.67 ± 30.80 | 103.92 ± 20.74 | 109.21 ± 31.42 | 118.88 ± 36.71 | <0.0001 |
| **UA(mg/dl)** | 4.63 ± 1.25 | 4.45 ± 1.19 | 4.54 ± 1.19 | 4.90 ± 1.31 | <0.0001 |
