# Supplementary material for: Association between atherogenic index of plasma, body mass index, and sarcopenia: a cross-sectional and longitudinal analysis study based on older adults in China
Source: Aging Clin Exp Res. 2025 Apr 7;37(1):122. doi: 10.1007/s40520-025-03029-0 (PMC11976801; doi:10.1007/s40520-025-03029-0)
Supplement: Supplementary file 2 — Supplementary Material 2 [file 40520_2025_3029_MOESM2_ESM.docx]

**Table S2 Baseline characteristics of study population by BMI**

|  | **Total(n=1441)** | **＜18.5(n=46)** | **18.5~23(n=532)** | **23~27.5(n=545)** | **＞27.5(n=318)** | **p.value** |
| --- | --- | --- | --- | --- | --- | --- |
| **Age** |  |  |  |  |  | <0.001 |
| 60-64 | 757(52.53) | 18(39.13) | 267(50.19) | 286(52.48) | 186(58.49) |  |
| 65-69 | 397(27.55) | 8(17.39) | 152(28.57) | 156(28.62) | 81(25.47) |  |
| ≥70 | 287(19.92) | 20(43.48) | 113(21.24) | 103(18.90) | 51(16.04) |  |
| **Gender** |  |  |  |  |  | <0.0001 |
| Female | 710(49.27) | 22(47.83) | 215(40.41) | 289(53.03) | 184(57.86) |  |
| Male | 731(50.73) | 24(52.17) | 317(59.59) | 256(46.97) | 134(42.14) |  |
| **Marital status** |  |  |  |  |  | <0.01 |
| Non-Married | 1226(85.08) | 32(69.57) | 444(83.46) | 473(86.79) | 277(87.11) |  |
| Married | 215(14.92) | 14(30.43) | 88(16.54) | 72(13.21) | 41(12.89) |  |
| **Education** |  |  |  |  |  | 0.01 |
| High school or above | 88( 6.11) | 2( 4.35) | 29( 5.45) | 43( 7.89) | 14( 4.40) |  |
| Illiterate | 428(29.70) | 23(50.00) | 146(27.44) | 158(28.99) | 101(31.76) |  |
| Junior high school or below | 925(64.19) | 21(45.65) | 357(67.11) | 344(63.12) | 203(63.84) |  |
| **Location** |  |  |  |  |  | <0.0001 |
| Rural | 917(63.64) | 40(86.96) | 382(71.80) | 321(58.90) | 174(54.72) |  |
| Urban | 524(36.36) | 6(13.04) | 150(28.20) | 224(41.10) | 144(45.28) |  |
| **Smoke** |  |  |  |  |  | <0.0001 |
| No | 1001(69.47) | 22(47.83) | 317(59.59) | 412(75.60) | 250(78.62) |  |
| Yes | 440(30.53) | 24(52.17) | 215(40.41) | 133(24.40) | 68(21.38) |  |
| **Drink** |  |  |  |  |  | <0.01 |
| No | 950(65.93) | 27(58.70) | 329(61.84) | 357(65.50) | 237(74.53) |  |
| Yes | 491(34.07) | 19(41.30) | 203(38.16) | 188(34.50) | 81(25.47) |  |
| **Hypertension** |  |  |  |  |  | <0.0001 |
| No | 948(65.79) | 35(76.09) | 427(80.26) | 344(63.12) | 142(44.65) |  |
| Yes | 493(34.21) | 11(23.91) | 105(19.74) | 201(36.88) | 176(55.35) |  |
| **Dyslipidemia** |  |  |  |  |  | <0.0001 |
| No | 1264(87.72) | 44(95.65) | 505(94.92) | 473(86.79) | 242(76.10) |  |
| Yes | 177(12.28) | 2( 4.35) | 27( 5.08) | 72(13.21) | 76(23.90) |  |
| **Diabetes** |  |  |  |  |  | <0.0001 |
| No | 1334(92.57) | 45(97.83) | 512(96.24) | 507(93.03) | 270(84.91) |  |
| Yes | 107( 7.43) | 1( 2.17) | 20( 3.76) | 38( 6.97) | 48(15.09) |  |
| **Chronic lung diseases** |  |  |  |  |  | 0.11 |
| No | 1271(88.20) | 36(78.26) | 478(89.85) | 480(88.07) | 277(87.11) |  |
| Yes | 170(11.80) | 10(21.74) | 54(10.15) | 65(11.93) | 41(12.89) |  |
| **Liver disease** |  |  |  |  |  | 0.16 |
| No | 1393(96.67) | 45(97.83) | 517(97.18) | 530(97.25) | 301(94.65) |  |
| Yes | 48( 3.33) | 1( 2.17) | 15( 2.82) | 15( 2.75) | 17( 5.35) |  |
| **Heart disease** |  |  |  |  |  | <0.001 |
| No | 1223(84.87) | 42(91.30) | 460(86.47) | 474(86.97) | 247(77.67) |  |
| Yes | 218(15.13) | 4( 8.70) | 72(13.53) | 71(13.03) | 71(22.33) |  |
| **Kidney disease** |  |  |  |  |  | 0.44 |
| No | 1358(94.24) | 45(97.83) | 499(93.80) | 518(95.05) | 296(93.08) |  |
| Yes | 83( 5.76) | 1( 2.17) | 33( 6.20) | 27( 4.95) | 22( 6.92) |  |
| **Stomach disease** |  |  |  |  |  | 0.22 |
| No | 1112(77.17) | 33(71.74) | 397(74.62) | 432(79.27) | 250(78.62) |  |
| Yes | 329(22.83) | 13(28.26) | 135(25.38) | 113(20.73) | 68(21.38) |  |
| **TC(mg/dl)** | 197.07 ± 38.23 | 202.02 ± 35.57 | 191.59 ± 38.28 | 198.88 ± 37.06 | 202.42 ± 39.50 | <0.001 |
| **HDL-C(mg/dl)** | 50.35 ± 14.71 | 63.06 ± 16.94 | 54.14 ± 15.29 | 48.65 ± 13.67 | 45.11 ± 12.23 | <0.0001 |
| **LDL-C(mg/dl)** | 120.61 ± 35.21 | 120.23 ± 29.27 | 115.92 ± 35.09 | 122.32 ± 34.07 | 125.59 ± 37.26 | <0.001 |
| **TG(mg/dl)** | 131.50 ± 95.98 | 99.27 ± 54.59 | 111.48 ± 85.94 | 138.83 ± 97.24 | 157.09 ± 106.08 | <0.0001 |
| **HbA1c(mg/dl)** | 5.34 ± 0.75 | 5.14 ± 0.36 | 5.21 ± 0.58 | 5.35 ± 0.78 | 5.56 ± 0.89 | <0.0001 |
| **CRP (mg/dl)** | 2.76 ± 6.05 | 2.10 ± 3.80 | 2.28 ± 5.02 | 2.83 ± 6.58 | 3.53 ± 6.81 | 0.03 |
| **GLU(mg/dl)** | 110.67 ± 30.80 | 103.59 ± 16.76 | 105.75 ± 24.15 | 111.17 ± 31.92 | 119.04 ± 37.76 | <0.0001 |
| **UA(mg/dl)** | 4.63 ± 1.25 | 4.43 ± 0.93 | 4.48 ± 1.20 | 4.70 ± 1.29 | 4.81 ± 1.27 | <0.001 |
